# Ingenious characterization and assessment of lentil germplasm collection to aphid *Acyrthosiphon pisum* stress unveils distinct responses

**DOI:** 10.3389/fpls.2022.1011026

**Published:** 2022-12-21

**Authors:** Ioannis Zafeiriou, Symela Ntoanidou, Eirini Baira, Konstantinos M. Kasiotis, Theodora Barmpouni, Kyriaki Machera, Photini V. Mylona

**Affiliations:** ^1^ Institute of Plant Breeding & Genetic Resources, Hellenic Agricultural Organization - DEMETER (HAO-DEMETER), Thermi, Greece; ^2^ Benaki Phytopathological Institute, Department of Pesticides Control and Phytopharmacy, Laboratory of Pesticides’ Toxicology, Athens, Kifissia, Greece

**Keywords:** *Lens culinaris*, aphids, hormones, defense genes, signaling pathways, metabolomics

## Abstract

Lentil cultivation is often hampered by aphid population outspreads with detrimental impacts to crop development and production, challenging food safety and agriculture sustainability. The pea aphid (*Acyrthosiphon pisum*) is a significant threat to lentil in the temperate zone rainfed systems. A set of management practices including resilient cultivars and application of insecticides have effectively controlled aphid infestation. However, the plant defense against insect pests is scantily dissected and limited to the individual components including antibiosis, antixenosis and tolerance that constitute a combination of plant stress responses. Utilizing a lentil germplasm collection, we assessed the antixenosis and aphid tolerance mechanisms in association to important morphological parameters. Physiological parameters including relative water content (RWC) measured at 24h and 48h post-aphid infestation revealed genotype-specific responses. The contents of key plant hormones including salicylic acid (SA), jasmonic acid (JA), abscisic acid (ABA) and indoleacetic acid (IAA) implicated in defense signal-transduction pathways were also determined in lentil accessions after aphid herbivory infestation. In parallel, the expression of hallmark defense genes governed by SA- and JA-signaling pathways at 24h and 48h post aphid herbivory revealed significant differentiation patterns among the accessions. An interplay of hormone crosstalk is unveiled that possibly governs defense responses and aphid resistance. Besides the metabolomic profiling of accessions under aphid herbivory indicated the indispensable role of key secondary metabolites accumulation such as flavonoids, alkaloids, phenolics and fatty acids as a front line of plant defense and a potential integration of hormone signaling pathways in metabolome reprogramming. Overall, the study presents a panorama of distinct lentil responses to aphids and a critical view of the molecular mechanisms implicated in lentil insect defense to further our insight and advance crop protection and breeding approaches in a climate changing environment.

## 1 Introduction

Lentil (*Lens culinaris* Medik) is a cool season, annual pulse crop, predominately self-pollinating (95%). Identified from the Upper Palaeolithic period in the Franchthi Cave in Peloponnese (South Greece) lentil is amongst the first domesticated crops to appear in the country. Lentil domestication is linked to the First Agricultural Revolution, referred also as the Neolithic agriculture featured as a wide-scale transition of many human cultures from a lifestyle of hunting and gathering to that of agriculture and settlement, making an increasingly larger population possible (Chahota et al., 2018; Polidoros et al., 2022). Lentil seeds are valued as a rich source of plant proteins and iron in human nutrition. In addition they are an important source of fiber, minerals, vitamins, antioxidants and with a low fat content lentils contribute significantly to the basic nutritional requirements of the modern human’s diet (Reddy, 2018; Sarker et al., 2018). Thus it is among the most popular pulses and could be considered a staple food in many parts of the world as the only source of protein, with global production reaching 5.9 million tons, harvested in 5.2 million hectares (FAOSTAT).

Through centuries, lentil is cultivated in most temperate zones of the world, mainly in rainfed agroecosystems underscoring its adaptability to the diversity of soils and climatic conditions. Due to its symbiotic N_2_ fixation, lentil cultivation comprises a key crop of poor soils and rotation systems promoting amelioration of soil-quality and providing nutritious seeds (Ram et al., 2018; Liu et al., 2019). Hence it is proposed as a most suitable crop for sustainable agriculture and food safety in marginal lands (Sarker et al., 2018). The total lentil cultivated area and production have drastically increased over the last decade, still the crop yield exhibited moderate increments (approx. 1.2 tones/ha), compared to other legume crops. Pathogens including pests have a pronounced role in yield losses due to the changing climate. While genomic research is delayed due to the large (4.3 GB) lentil genome size, progress towards the release of the complete lentil genome sequence is expected to accelerate breeding efforts and molecular tools that are less developed in comparison to other crops (Polidoros et al., 2022).

Among the herbivorous insects, aphids occupy a prominent position of legume pests due to their aggregate destructive impacts. The pea aphid *Acyrthosiphon pisum* is a considerable plant pathogen able to exert a number of devastating impacts on yield and seed quality of legume crops including lentil. *A. pisum* feeds on plant sap, disrupting the normal plant growth pattern including reduction in root and nodulation growth. At high aphid densities plants are deformed, stunted and seed set is reduced. Climate-change and global warming adversely affect lentil cropping systems, especially in drought-prone regions of the temperate zone, like the Mediterranean basin. Increased temperatures and altered precipitation patterns have significant impacts on lentil production and on insect pests. Specifically changes in climate can result in an increased number of aphid generations, increased survival during overwintering, expansion of their geographic distribution, increased incidence of aphid-transmitted plant diseases, and reduced effectiveness of biological control, especially natural enemies (Skendžić et al., 2021).

The pea aphid has key role in the transmission of major destructive virus pathogens worldwide, as it can transmit over 40 plant pathogenic viruses among legume crops. Some of the main viruses transmitted by the pea aphid include the bean leafroll virus (BLRV), the pea enation mosaic virus (PEMV-1), the faba bean necrotic yellows virus (FBNYV), the soybean dwarf virus SbDV, and the beet western yellows virus (BWYV) with detrimental impact to legumes production (Rashed et al., 2018). Transmission of viruses from diseased to healthy plants within fields, and across neighboring fields results in severe virus epidemics and crop damages. Studies have shown that virus epidemics is often due to high aphid populations that spread the virus during their short feeding periods. Surveys conducted in many countries around the globe during the last three decades demonstrated that significant limitation to legume production including lentil, is caused by aphid-borne viruses (Pérez de la Vega et al., 2022).

Thus far, aphid population management relies mostly on prophylactic application of insecticides to properly secure the lentil crop production. However, global warming drivers counting increased temperatures and decrease in relative humidity may cause synthetic insecticides and management approaches to be less effective. Also the use of synthetic pesticides further exacerbates the carbon and energy footprint and the environmental impact raising concerns over field-evolved resistance of aphid populations to chemical formulations (Bhusal et al., 2021; Singh et al., 2021).

Knowledge of the lentil defense mechanisms to aphid herbivore is scarce impeding the development and breeding of resistance cultivars and of integrated pest management methods. This entails for a thorough understanding of the aphid stress induced plant defense responses and mechanisms. The plant defense against insect pests is a combination of three distinct components, antibiosis, antixenosis and tolerance (van Emden, 2017). Antibiotic traits interfere negatively with the insect’s biology and result in reduced growth, longevity and fecundity, and increased mortality. Antixenotic mechanisms influence the preference and the choice of a host by the insects, as well as their feeding behavior. Tolerance attributes reduce the impact of herbivory on the plants’ fitness in such a way, that the plant can withstand or recover from damages, while sustaining insect populations similar in size to those of susceptible plants. Thereby, there is no interference with the insects’ physiology *sensu stricto* and no selective pressure is exerted on the insect’s population, resulting in limited threat of virulent-biotype emergence (Nalam et al., 2019). To date a thorough assessment of these mechanism in lentil germplasm is scarce, limited to the assessment of particular aphid mechanism towards the selection of tolerant varieties.

Again, plants as sessile organisms have evolved a network of defense responses and molecular mechanisms to restrain the entrance of pathogens and the damage induced. Studies on model plants have shown that plant responses to herbivores including aphids are regulated by an interplay of phytohormones. Indeed, plants withstand herbivore attack by specific recognition of the attacker followed by hormones activating signal transduction pathways. In this context salicylic acid (SA), jasmonic acid (JA) and ethylene (ET) emerge as key regulators. Similarly, auxins such as indole-3 acetic acid (IAA) that regulate a vast array of plant processes including growth and development as well as responses to abiotic stress and pathogens were shown to have a primary role. Specifically, IAA is rapidly elicited by herbivore oral secretions and fatty acid conjugate elicitors and is accompanied by a rapid transcriptional increase of auxin biosynthesis genes. Studies in *Nicotiana attenuata* model plant have shown that IAA spreads rapidly from the aphid induced wound site to systemic tissues in a process completed prior to the JA burst and signaling pathway (Machado et al., 2016). Yet, the role of phytohormones in lentil-aphid interaction is overlooked.

On the other hand, recent investigation on fungal lentil diseases such as Ascochyta blight (*Ascochyta lentis)* has provided a breadth of information of the hormone defense signaling pathways and the pathogenesis related genes and proteins, known as PR, among resistant and susceptible lentil lines (Sari et al., 2017). *PR-1* and *PR-5* have been widely accepted as hallmarks of SA signaling pathway and *PR-4* and *AOC* genes as hallmarks of the JA signaling after ascochyta bight infection in lentil. Allene oxidase cyclase (*AOC*) is a key enzyme involved in JA biosynthesis from a-linolenic acid (Vick & Zimmerman, 1984) and has potential utility as a marker for monitoring the JA signaling pathway.

Apart of plant hormones the plant arsenal of low molecular weight organic compounds encompasses a variety of substances that are separated by the perspective function into primary and secondary metabolites. This diversified synthetic capacity of plants has evolved to support their static *modus vivendi* and adaptation to adverse environments counting abiotic and biotic stresses. Primary metabolites are ubiquitous in all plants and play crucial housekeeping roles in plant growth and development (Fàbregas & Fernie, 2021). Secondary metabolites mediate plant–environment interactions, while recent genetic and chemical evidence demonstrated that entwined in various physiological and biochemical processes including development, growth and defense regulation (Erb and Kliebenstein, 2020). Based on their core structure secondary metabolites form three major categories: nitrogen-containing chemical compounds, terpenoids and phenolics. The nitrogen-containing compounds consisting of alkaloids, glucosinolates and cyanogenic glucosinolates have been widely identified in plants. Terpenoids encompass a number of subgroups on the basis of the number of isoprene structural units (i.e., monoterpenes, sesquiterpenes, diterpenes, triterpenes etc.). Phenolics engulf compounds containing at least one aromatic ring and one hydroxyl moiety, such as phenolic acids, flavonoids, tannins, etc. (Zhan et al., 2022). Several studies showed that plant secondary compounds have detrimental effects on pea aphids (Züst & Agrawal, 2016). For example, higher levels of saponins and phenolic compounds led to the reduction of aphid population growth. Sanchez-Arcos et al. conducted the metabolic fingerprinting of *Medicago sativa*, *Trifolium pratense*, *Pisum sativum* and *Vicia faba* under infestation with native and non-native aphid species races demonstrated that differences in the flavonoids, saponins, non-proteinogenic and peptides profile are plant species-related and aphid race-related (Sanchez-Arcos et al., 2019). Interestingly, hormone differences might lead to changes in plant metabolomes, especially for compounds having a deterrent or toxic impact on aphids. Thus, steroidal and triterpene saponins were specific and also the most abundant classes in *M. sativa* aphid infested plants. While alterations in their phenolic compounds’ content also demonstrated various effects in the feeding behavior of the pea aphid (Yan et al., 2018).

The present study constitutes a comprehensive multi-level assessment and characterization of lentil germplasm to aphid infestation. To our knowledge this is the first study that dissects aphid infestation at various levels using a collection of lentil accessions aiming to thoroughly scrutinize the lentil, genotype responses. More specifically, we evaluated the antixenosis and tolerance performance of 16 lentil genotypes including landraces and cultivars. A set of growth parameters were assessed to determine the impact of aphid infestation. Analysis of endogenous plant hormones counting SA, JA, IAA and ABA and the expression of hallmark genes implicated in the SA and JA pathways were also examined. The impact of aphid infestation and herbivory on secondary metabolites profile was further assessed contributing to a panorama of differential lentil responses.

## 2 Materials and methods

### 2.1 Plant material and growth conditions

**Figure 1:**
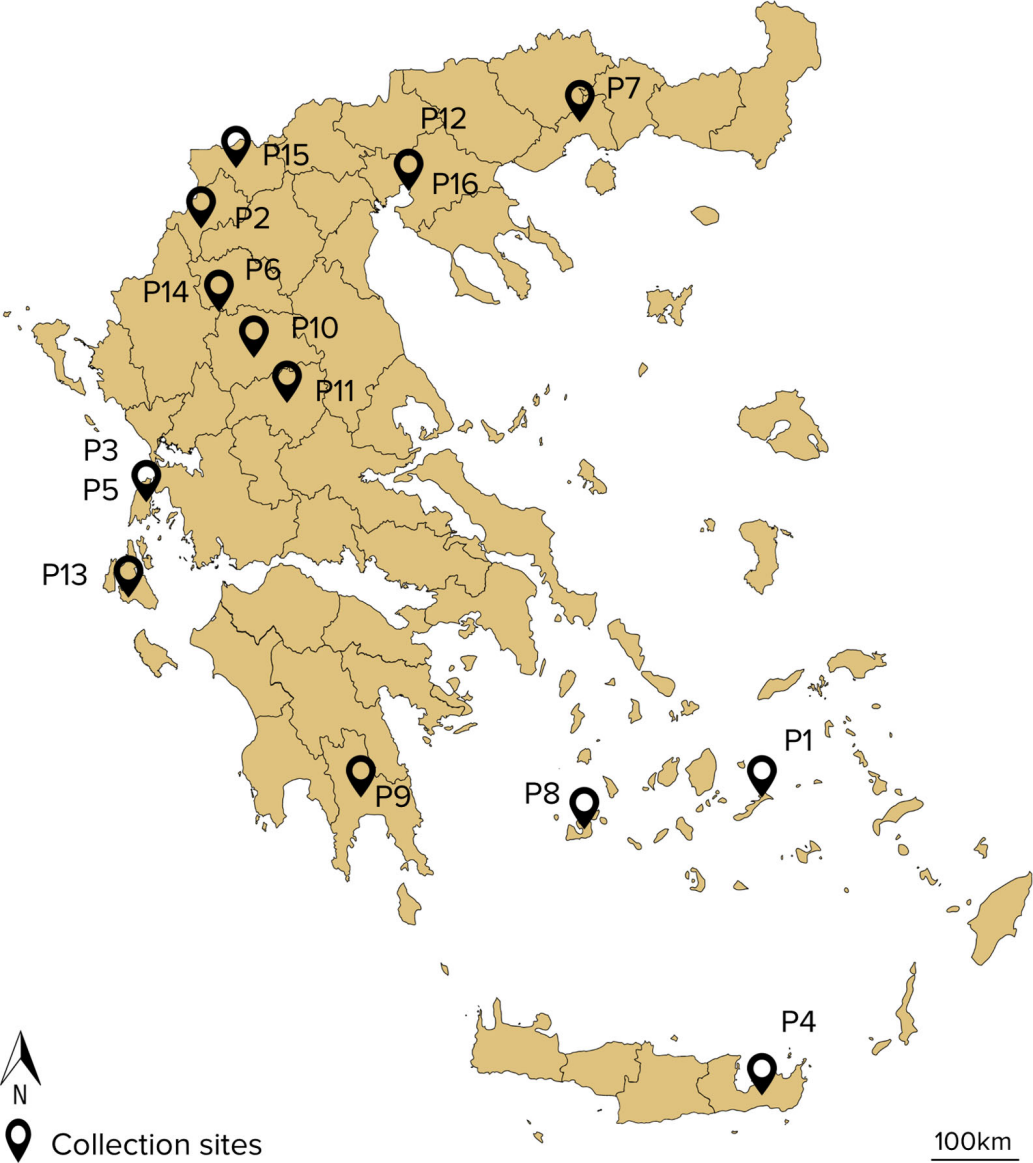
Map of the collection sites of lentil accessions used in the study (Google, n.d.).

The lentil collection of modern cultivars and landraces was obtained from the Institute of Plant Breeding & Genetic Resources (IPB&GR) of the Hellenic Agricultural Organization-DEMETER. The lentil collection of 16 accessions comprised of 14 local landraces and 2 modern cultivars that originated from Greece (**Table 1**). **Figure 1** depicts the collection site of the landraces. Twenty seeds of each accession were sown in 4:1 peat:perlite, 2L pots and allowed to germinate and grow in semi-field conditions at IPB&GR in Thessaloniki, Greece. Seedlings of similar size and appearance at the developmental stage V6-V7 (Erskine et al., 1990) were used for all the subsequent bioassays.

**Table 1:** Details on the collection sites and origin, genotype and sample source of the plant material.

Code	Accession	Collection Site/Origin	Territory	Genotype
P1	ANP-065	Amorgos	Island	Landrace
P2	F-173	Kastoria	Mainland	˝
P3	GRC-0007	Lefkada, Egklouvi	Island	˝
P4	HL-164	Lasithi	˝	˝
P5	IK-029	Lefkada, Karies	˝	˝
P6	K-216	Grevena	Mainland	˝
P7	KD-101	Kavala	˝	˝
P8	MFS-062	Milos	Island	˝
P9	P-118	Lakonia	Mainland	˝
P10	T-452	Trikala	˝	˝
P11	T-465	Karditsa	˝	˝
P12	DEMETRA	Greece	˝	Cultivar
P13	IK-100	Kefalonia	Island	Landrace
P14	K-169	Grevena	Mainland	˝
P15	K-221	Florina	˝	˝
P16	SAMOS	Greece	˝	Cultivar

*According to the European Plant Variety Database (PVD, 2021).

### 2.2 Aphid population culture

For the establishment of *Acyrthosiphon pisum* colonies, seeds of every lentil accession were sown in 2L pots. Soon after the germination, the seedlings were infested with aphids of various developmental stages, identified and collected from the adjacent lentil-crop fields. The infested plants were isolated in mesh boxes, under greenhouse conditions. All plants were watered as required and apterous aphids of these primary colonies were used for all the following experimental trials.

### 2.3 Host-preference experiment

The antixenosis test was performed based on Webster’s method with some modifications (Webster, 1990). Sixteen seedlings (one per landrace) were sown in a 10L pot, arranged in a ~24cm diameter circle, with a 4.5cm interval space between neighboring seedlings. A petri dish containing 180 adult, apterous individuals, was placed at the center of the pot, providing equal chance for every different accession to be selected by the aphids. The insects were released in the evening, in order to avoid phototaxis effect (Y. Zhang et al., 2016). The pot was covered with linen mesh, supported by a bamboo stick, providing adequate air and lighting. The number of aphids on every seedling was counted after 48h (48hpi). The experiment was conducted in a randomized complete block design (RCBD) with 10 independent replications, under greenhouse conditions, with photoperiod 16/8h, day temperature 18-20^o^C and night temperature 14-16 ^o^C.

### 2.4 Tolerance test

Sixteen lentil accessions were tested for tolerance to *A. pisum*. Five seedlings of every accession, at the V6 stage (18.3cm average height) were divided in two different pots, set side-by-side, in order to be exposed in the same environmental conditions. For every accession, one pot was infested with aphids (10 adult, apterous individuals per plant), while the other remained noninfested, serving as experimental control. Care was taken to maintain a constant number of 10 aphids per plant, by removing newborn aphids and by adding new mature individuals when needed. All pots were covered with linen mesh, in order to restrain the aphids within the treated-pot and prevent any potential contamination of the control group. Tolerance was quantified by recording the aboveground fresh weight (FW), dry weight (DW) and height (H) before the infestation, as well as for 12 days after the infestation (12dpi). The tolerance experiment was carried out once, in a randomized complete block design (RCBD) with five replications per accession, under greenhouse conditions, with photoperiod 16/8h, day temperature 18-20^o^C and night temperature 14-16 ^o^C. All plants were irrigated once per week, with the same amount of water, while no fertilization was applied. The plant FW (a), DW (b) and H (c) reduction of every accession were measured according to the following formulas (Reese et al., 1994):


$$FW\;reduction\;\left(\%\right)=\frac{FW\;of\;the\;control\;plant-FW\;of\;the\;infested\;plant}{FW\;of\;the\;control\;plant}\;x\;100$$

$$DW\;reduction\;\left(\%\right)=\frac{DW\;of\;the\;control\;plant-DW\;of\;the\;infested\;plan}{DW\;of\;the\;control\;plant}\;x\;100$$

$$H\;reduction\;\;\left(\%\right)=\frac{H\;of\;the\;control\;plant-H\;of\;the\;infested\;plant}{H\;of\;the\;control\;plant}\;\;x100$$

### 2.5 Expression analysis of genes involved in the SA and JA signal transduction pathways

The three upper leaves on the main stem, of three individual plants per accession, were infested with ten apterous aphids, and covered with linen mesh. Infested plant tissue samples were collected at 0hpi, 24hpi, and 48hpi and stored at -80^o^ C for subsequent RNA assays. Leaf total RNA was extracted using Monarch^®^ Total RNA Miniprep Kit Protocol Cart (NewEngland Biolabs, Ipswich, MA), according to the manufacturer’s protocol. Qualitative and quantitative check was performed by NanoPhotometer P330 (Implen, Munich Germany) and visualized on 2% agarose gel electrophoresis. The primers used (Vaghefi et al., 2013; Sari et al., 2017) for the relative genes expression assessment are shown in **Supplementary Table 1**. The temporal pattern of SA and JA signaling after aphid (*A. pisum*) infection was indirectly assessed by expression analysis of *PR-1 *and *PR*-*5* as hallmarks of the SA pathway and *PR-4* and *AOC* as hallmarks of the JA pathway. *AOX* was used as an indicative gene of oxidative stress response and cellular redox balance to biotic adversities and a putative mediator of the phytohormone changes associated to piercing-sucking insects (Zhang et al., 2012).

The RT-qPCR was performed using Luna^®^ Universal One-Step RT-qPCR Kit (NewEngland Biolabs, Ipswich, MA) in a total volume of 20μL reaction mix, containing 10μl of Luna Universal One-Step ReactionMix 1X, 0.4μM of each primer and 1U Luna WarmStart RT Enzyme Mix. The reactions were carried out on LightCycler 96 (Roche Diagnostics Gmbh, Mannheim, Germany), and the RT-qPCR conditions include: reverse transcription for 10min at 55^o^C, initial denaturation for 1min at 95^o^C, and 40 cycles of denaturation for 10sec at 95^o^C, and extension for 30sec at 58^o^C. The melting curve analysis includes denaturation for 60sec at 95^o^C, annealing for 60sec at 40^o^C, and a final denaturation at 60^o^C for 1sec and at 95^o^C for 1sec. Three technical replicates of each sample were included in the RT-qPCR. The β-actin housekeeping gene from *Lens culinaris* (Medik) was used as endogenous reference. The gene expression levels were determined by normalizing the data to the reference gene *LcActin-257* (Sari et al., 2017). The normalized gene expression data were analyzed by the 2^-ΔΔCt^ method (Livak and Schmittgen, 2001) and were reported relative to the 0hpi. Then the 2 ^–ΔΔCt^ values of every transcript were expressed by the log_2_ formula and were used for the visual representation of relative gene expression on a heatmap, using TBtools (Chen et al., 2020).

### 2.6 Leaf relative water content as a guideline for stress response

Leaves of the same developmental stage were collected from seedlings infested with 10 aphids at three distinct time points: 0hpi, 24hpi, and 48hpi. For every sample, the leaves were let float in distilled water for 3h and the fresh weight at full turgor (TW) was measured. Then the samples were incubated at 55^o^C for 24h in order to measure the DW. The experimental design was completely randomized with three independent replications per accession and timepoint. For the calculation of the Relative Water Content (RWC) we used the formula below (Claussen, 2005):


$$RWC=\frac{FW-DW}{TW-DW}$$

### 2.7 Phytohormone analysis

Harvested tissues were immediately frozen in liquid nitrogen and stored at -80°C until analysis. Approximately 50mg of leaf and stem tissue was weighed in a 1.5mL Eppendorf tube. After the addition of 1 mL ice-cold 50% (v/v) aqueous acetonitrile (ACN), the sample was homogenized-extracted with a TissueLyser LT (QIAGEN, Hilden, Germany) for 4 min at 25Hz using one stainless steel bead (5mm, QIAGEN, Hilden, Germany). The extract was then centrifuged for 5min at 4,500 rpm at 4°C (Heraeus Labofuge 400R, Thermo Scientific, Germany). The supernatant was then purified using reversed-phase solid-phase extraction (RP-SPE) Oasis HLB cartridge (3mL Vac Cartridge, 60mg sorbent per cartridge, 30µm, Waters, Auckland, New Zealand). The column was activated with 1mL 100% methanol and 1 mL ultrapure water, and equilibrated with 1mL 50% (v/v) aqueous ACN. The sample was loaded onto the cartridge and the eluate was collected. The residues of the target compounds were then eluted with 1mL of ice-cold 30% (v/v) aqueous ACN. The eluate was collected, filtered (Nylon filter), and directly injected into the LC-ESI-MS/MS system.

Abscisic acid (ABA), salicylic acid (SA), jasmonic acid (JA) and 3-indoleacetic acid (IAA), were of analytical standards, with purity above 95% and were purchased from Supelco (Bellefonte, PA, USA). Methanol (MeOH) and acetonitrile (ACN) were of LC-MS grade, ammonium acetate, formic acid and acetic acid, all obtained from Merck (Darmstadt, Germany). Ultra-pure water was produced from SG Milipore apparatus. Nylon filters (0.22μm) were obtained from Macherey-Nagel (Dueren, Germany).

### 2.8 Liquid chromatography electrospray tandem mass spectrometry

For the mass spectrometric analysis an Agilent Technologies 6410 Triple Quad LC/MS system was used. The chromatographic separation was achieved by injecting 10μL of sample on a di-phenyl column (Fortis Diphenyl 2.1 x 150mm, 3.0μm) thermostated at 40°C. The mobile phase consisted of, channel α: water containing 0.1 % formic acid, 0.3mmol/L ammonium acetate adjusted to pH 4.0 with acetic acid, and channel β: 90% aqueous acetonitrile, 0.1% formic acid, 0.3mmol/L ammonium acetate, adjusted to pH 4.0 with acetic acid, using a flow rate 0.3mL/min and a gradient. The mass spectrometer was operated in Multiple Reaction Monitoring (MRM) mode using both positive and negative Electron Spray Ionization (ESI). Nitrogen was used as nebulizer (pressure at 40psi) and collision gas. The source gas temperature was set at 300°C, with a gas flow of 11L/min. For instrument control, Agilent Mass Hunter data acquisition Triple Quad B.01.04 and for data processing Agilent MassHunter Workstation Qualitative Analysis B.01.04. were used.

### 2.9 High-resolution mass spectrometry analysis

Approximately 50mg of leaf tissue was weighed in a 1.5mL Eppendorf tube. After the addition of 400μL of a mixture of MeOH:H_2_O (4:1, v/v) containing 0.5% formic acid, the mixture was vortex shaken for 30sec (2200μL/min, MS1 Minishaker IKA, Staufen, Germany) and was left for 40sec in an ice-bath (4°C). The sample was then homogenized-extracted with a TissueLyser LT (QIAGEN, Hilden, Germany) for 4min at 25Hz assisted by one stainless steel bead (5mm, QIAGEN, Hilden, Germany) positioned inside the Eppendorf tube. The extract was then centrifuged (Heraeus PICO17, Thermo Scientific, Germany) for 10min at 12,000rpm (ambient temperature). Consequently, the supernatant was transferred to another equivalent Eppendorf tube and subjected to additional centrifugation (5min, 12,000rpm, ambient temperature). The final supernatant was collected, filtered (Nylon syringe filter 0.22μm (Macherey Nagel, Dueren, Germany), and directly injected into the UHPLC-HRMS/MS system.

### 2.10 Orbitrap high resolution mass spectrometry analysis

For the investigation of the chemical profiling of the extracts a Q-Exactive Orbitrap platform (Thermo Fisher Scientific, San Jose, CA, USA) was employed. The samples were analyzed using a Hypersil Gold UPLC C18 (2.1×150mm,1.9μm) reversed phased column (Thermo Fisher Scientific, San Jose, CA, USA) in positive (ESI+) and negative (ESI-) ion modes. Eluent A (ultrapure water with 0.1% formic acid) and B (acetonitrile with 0.1% formic acid) were used in a gradient mode of 30min as follows: 0 to 21min: 5% B, 21 to 24min: 95% B, 24 to 30min: 5% B. The flow rate was 0.22mL/min and data acquisition was performed on a mass range of 120–1300Da on centroid mode. The conditions for HRMS for negative and positive modes were set as follows: capillary temperature, 320^°^C; spray voltage, 3.6kV (for positive mode) and 2.7kV (for negative mode); S-lens Rf level, 55V (for positive mode) and 50 (for negative mode); sheath gas flow, 40 arb. units; aux gas flow, 8 arb. units; aux. gas heater temperature, 200°C. The resolution for full scan analysis was set on 70,000 whereas for the data dependent acquisition mode the resolution was 35,000 allowing for MS/MS fragmentation of the three most intense ions. Stepped normalized collision energy was set at 35, 60, and 100. The column temperature was kept at 40°C while the sample tray temperature was set at 4°C. After the acquisition, the data were processed using the Compound Discoverer version 2.1 (Thermo Fisher Scientific, San Jose, CA, USA) for peak detection, deconvolution, deisotoping, alignment, gap filling and composition prediction procedures.

Statistical analysis was performed using Simca software for Principal Component Analysis (PCA) and Partial least squares-discriminant analysis (PLS-DA) to explore the existence of possible clustering formation. Permutation testing was performed employing 100 random permutations. The variables with ratio (treatment *vs* control) >1.5 and<0.667 and adjusted p-values (Benjamini-Hochberg) ≤0.05 were considered as contributing the most to group separation and forward for annotation using *in house* and online databases such as mzCloud, Metlin, LipidMaps, Dictionary of Natural Products, taking into consideration the isotopic and MS/MS fragmentation pattern, when it was applicable, and applying *m/z* tolerance of ±5 ppm.

### 2.11 Statistical analysis

In order to test whether there are statistically significant differences among the accessions regarding the aphid-feeding preference, the FW, DW, H reduction, the RWC, the gene expression, the abundance of phytohormones and secondary metabolites after aphid infestation and the assignment to different groups, we performed Multivariate Analysis of Variance (MANOVA), Analysis of Variance (ANOVA) and Tukey’s HSD test (p≤0.05). Possible correlation of the antixenosis effect of the accessions to their geographical origin was tested using Mantel’s test. Clustering of the accessions regarding their relative gene expression was executed by Principal Component Analysis (PCA). All the aforementioned analyses were conducted with R software version 4.2 (R Core Team, 2021).

## 3 Results

### 3.1 Host-preference experiment

Forty-eight hours (48h) after the release of 1800 aphids in 16 lentil accessions, only 1224 (approx. 68%) individuals were recovered. The antixenosis data analysis revealed that the aphid colony size was significantly affected by the lentil accession, genotype, under evaluation (*p<0.05*). The cultivar with the highest preference rate of aphids was P16 (12.13 insects per plant), followed by P15 (10.25 insects per plant). The least preferred genotype was P2 (2.25 insects per plant), followed by P3 (3.25 insects per plant) and P13 (3.38 insects per plant) (**Table 2**). A moderate correlation among the antixenosis performance of the accessions and their geographical site of origin was revealed by Mantel’s test (R=0.445, *p<0.001*).

**Table 2:** Mean colony size of *A. pisum* on 16 lentil accessions, measured as mean number of aphids on each genotype.

Accession	Mean colony size
P1	6.63^cdef^±0.7	
P2	2.25^a^±1.8	
P3	3.25^ab^±1.3	
P4	7.75^def^±1.5	
P5	4.38^abc^±1.5	
P6	8.13^efg^±2.3	
P7	5.13^bc^±0.8	
P8	8.25^efg^±0.9	
P9	8.75^fg^±1.9	
P10	4.38^abc^±1.3	
P11	5.63^bcd^±0.9	
P12	8.25^efg^±1.5	
P13	3.38^ab^±1.3	
P14	6.25^cde^±0.5	
P15	10.25^gh^±1.2	
P16	12.13^h^±1.7	

*Mean colony sizes that share no common letter are statistically significant at a=0.05.

The accessions that are the least preferred by the aphids, originated from Lefkada (P3, P5) and Kefalonia (P13) islands in western Greece and from central (P10, P11) and (P2) from northern Greece. The commercial cultivar P12 antixenosis performance is similar to many landraces, and attracts a significantly lower number of aphids, compared to cultivar P16. According to genomic studies, both cultivars share common ancestry, however in the recently developed cultivar P16, the antixenosis effect might has been abolished (Tsanakas et al., 2018).

### 3.2 Tolerance test

**Figure 2:**
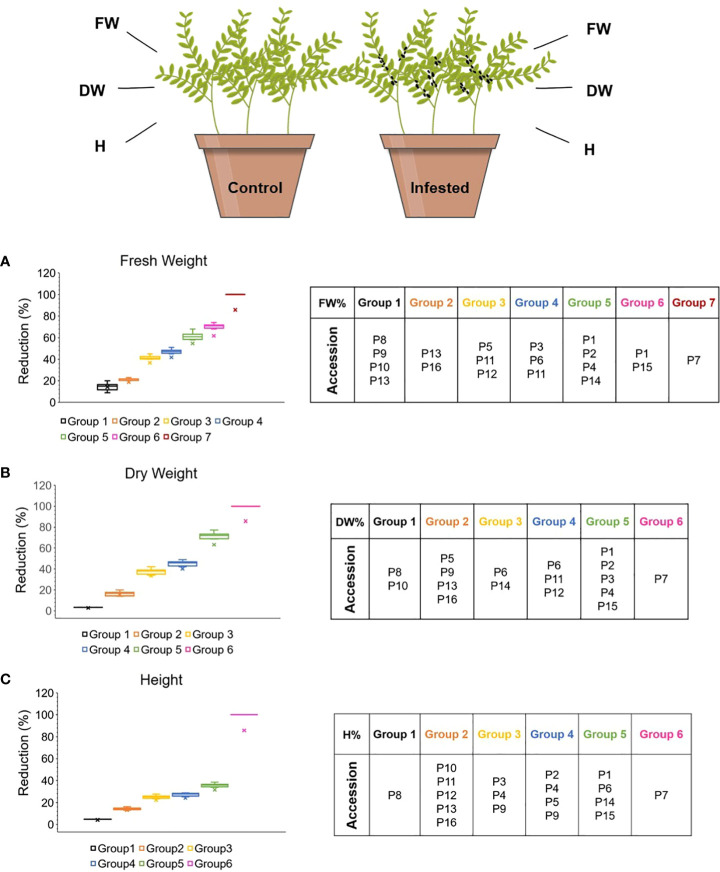
Effect of aphid herbivory on the **(A)** FW, **(B)** DW, **(C)** H reduction (%) of the different lentil accessions 12dpi. The accessions were assigned to different groups based on their post infestation performance, according to Tukey’s HSD (*p*<0.05). The figure was created with BioRender.com.

Decreased growth is a common plant defense response to the imposition to biotic stress. Resilient plants can support large insect populations, to the detriment of little damage or yield loss. The response of the 16 lentil accessions to the colonization with *A. pisum* was investigated by measuring their aboveground fresh weight (FW), dry weight (DW) and height (H), both prior and after aphid infestation, as well as their percent reduction. According to Tukey’s HSD test (*a=0.05*) the accessions were distributed in groups of similar performance regarding the FW%, DW% and H% reduction at 12dpi, depicted in 7, 6 and 6 groups, respectively (**Figures 2A–C**). The infestation with aphids had a significant effect in the reduction of FW, DW (up to 80%) and H (up to 35%) in all accessions, except for the DW reduction in P8 and P10 (*p<0.05*) (**Supplementary Table 2**).

Interestingly landrace P7 did not survive after aphid colonization for 12 consecutive days (FW, DW, H reduction 100%). Regarding the commercial cultivars’ performance, P16 demonstrated significantly lower FW and DW reduction (23% and 20%, respectively) than P12 (41% and 49%, respectively). Noting that for every different variable, the groups composition may be varied. A two-tailed Pearson’s correlation revealed significant strong positive correlation between the plant FW and DW (R=0.923), FW and H (R=0.817) and DW and H (R=0.679) reduction (*p*<0.01) (**Supplementary Table 3**).

### 3.3 Analysis of the SA and JA signal transduction pathways by RT-qPCR

To further discern the interplay of phytohormones in defense signaling pathways that deploy defense responses we assessed a set of hallmark genes expression patterns that are known to be triggered either through SA or JA-mediated signaling pathways in response to pathogens and insects. As previous knowledge of lentil gene defense expression patterns in response to feeding aphids is lacking, we assessed the expression of the *PR-1, PR-4* and *PR-5* genes. *PR-1* and *PR-5* are hallmarks of the SA pathway and *PR-4* and *AOC* are considered hallmarks of the JA and ABA pathway. Along with these the alternative oxidase (*AOX*) gene expression was assessed as an indicative gene of stress response and cellular redox balance to biotic adversities.

**Figure 3:**
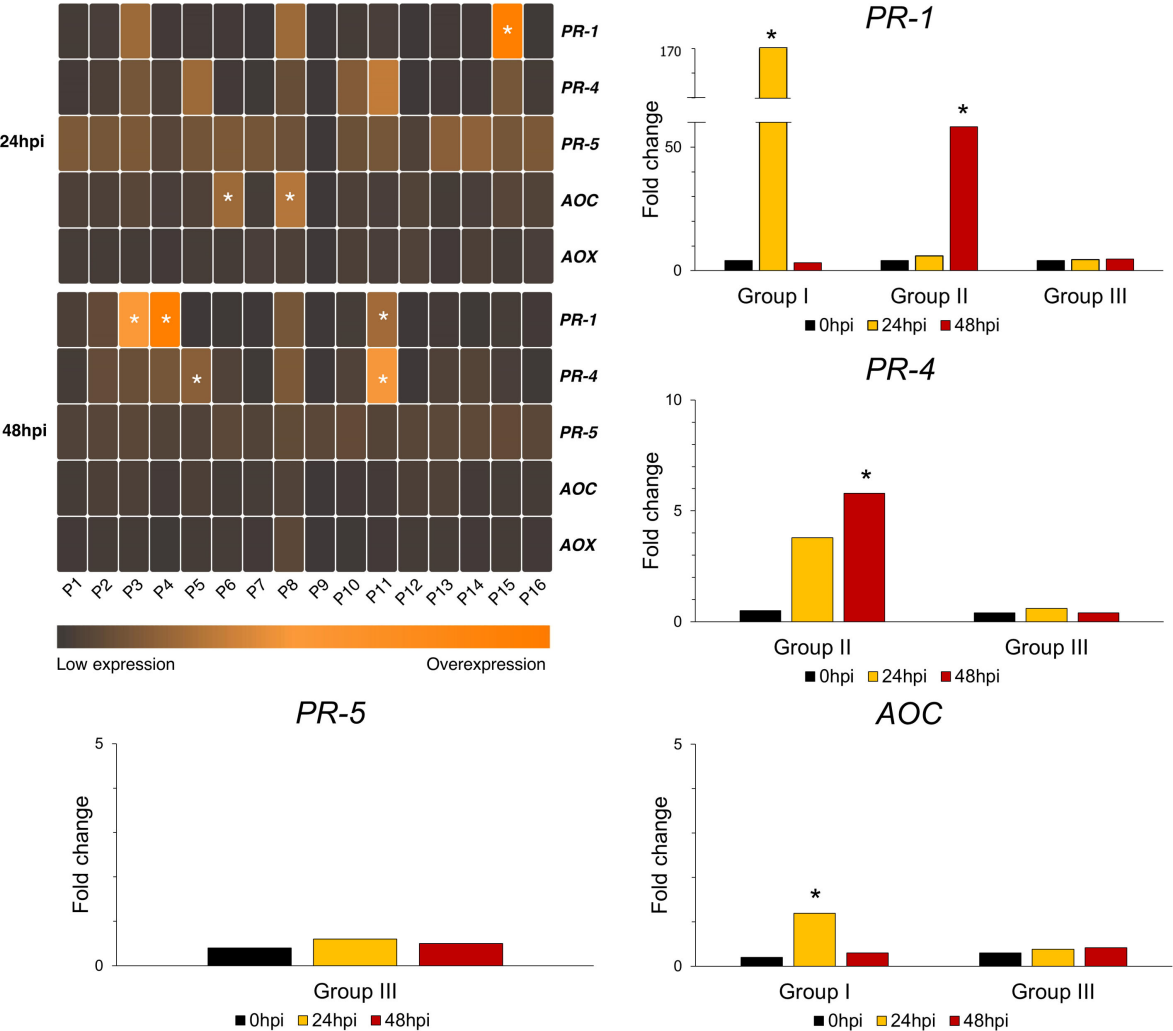
Differential expression heatmap of the genes involved in SA (*PR-1*, *PR-5*), JA (*PR-4*, *AOC*) and Alternative Oxidase (*AOX*) pathways, relative to 0hpi. According to Tukey’s HSD (*a=0.05*), genotypes of similar gene expression performance are arranged to the same group. For every different gene, the groups may consist of different accessions. In the heatmap, the colored cells indicate the presence of a group. The performance of the different groups is also depicted in histograms as log2 transformed expression levels. The asterisk (*) indicates statistically significant differences (p<0.05).

Upregulated gene expression was detected 24hpi for *PR-1*, that did further increase in P3, P4 and P11, but declined for the rest of the accessions 48hpi. The PR-5 transcription levels were elevated in all accessions 24hpi and were subsequently reduced, apart from P9 that demonstrated increased expression only at 48hpi. Regarding PR-4, solely accessions P3, P4, P5, P8, P10, P11 and P15 showed increased expression levels since 24hpi, whereas all accessions had increased transcription levels at 48hpi. The AOC expression was upregulated 24hpi compared to control, and was further declined. The expression levels of AOX remained similar to those of control. The t-test analysis revealed significant differences in the genes’ expression at 24hpi and 48hpi, according to which the accessions were arranged in three different groups. In particular, three different groups were formed (**Figure 3**). Group I consists of accessions that demonstrated significant upregulation of the gene expression at 24hpi, whereas Group II includes the accessions with significantly increased gene expression at 48hpi. The accessions that didn’t change their gene expression significantly after aphid infestation are included in Group III. Details on the expression pattern of the aforementioned genes are provided in (**Supplementary Table 4**).

A Principal Component Analysis (PCA) based on the relative expression of all the examined genes, revealed that all accessions were clustered together in the first two axes (60.16%), except for P9 (**Supplementary Figure 1**). Landrace P9 exhibited a decrease in transcript abundance of all genes examined, both after 24h and 48h of aphid infestation.

### 3.4 The leaf relative water content marker

**Figure 4:**
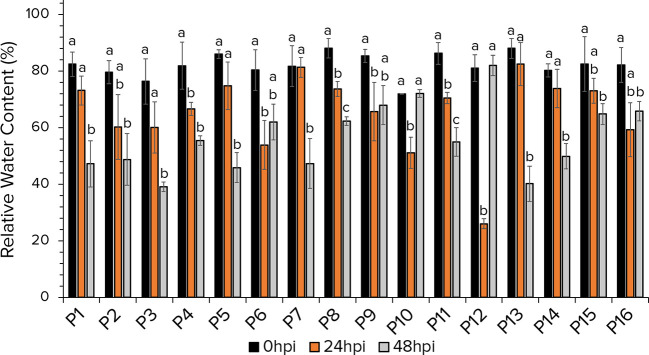
The leaf Relative Water Content of 16 lentil accessions at 0hpi, 24hpi and 48hpi with aphids. For each accession, estimates at different timepoints that share no common letter are statistically significant according to Tukey’s HSD (*p*<0.05).

The leaf RWC of aphid infested lentil plants revealed significant fluctuations (**Figure 4**). In most of the accessions, reduction in the RWC was detected at 48 hours after aphid herbivory, while only in accessions P6, P9, P10 and P12 the RWC decreased at 24hpi. Remarkably accessions P6, P9, P10, P12 and P16 the RWC at 48hpi is higher that that at 24hpi, indicating the possible implication of secondary metabolites induction to support the plant’s functionality intruded by aphid infestation. While the RWC at 48hpi in accessions P10 and P12 recovered to control levels.

### 3.5 Phytohormonal profiling of aphid-infested lentil germplasm

**Figure 5:**
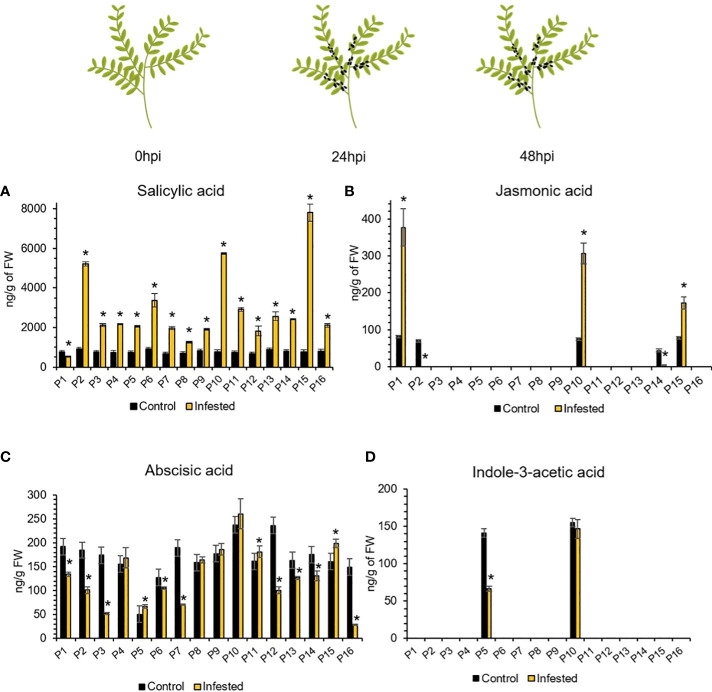
Relative abundance of **(A)** SA, **(B)** JA, **(C)** ABA, and **(D)** IAA at 48hpi compared to 0hpi. Accessions of similar phytohormonal abundance between control and 48hpi are considered unchanged according to Tukey’s HSD (*a=0.05*). The asterisk (*) indicates statistically significant differences (*p<0.05*). Omitted bars indicate values below the limits of quantification (LOQs) of the method.

To examine if *A. pisum* feeding elicits changes in the phytohormonal levels of lentil plants that trigger defense signaling pathways and deploy defense, we determined the contents (ng/g of FW) of salicylic acid (SA), jasmonic acid (JA), abscisic acid (ABA) and indole-3-acetic acid (IAA) in the aphid herbivory treated plants at 48hpi and in control plants, independently in the 16 accessions of the lentil collection. The phytohormone contents of the treated and control accessions was investigated by Liquid Chromatography-tandem Mass Spectrometry (LC/MS-MS) analyses. Overall, the approach allowed to annotate four phytohormones, namely SA, JA, ABA and IAA, that are known to participate in plant-biotic stress responses at 48hpi. The ANOVA revealed significant fluctuation of the abundance of the phytohormones prior and after 48h of herbivory (*p<0.001*). The SA content increased significantly at 48hpi in all accessions, except for P1 (**Figure 5A**). The ABA content was elevated in P5, P11 and P15 and reduced in P1, P2, P3, P6, P7, P12, P13, P14 and P16. Accessions P4, P8, P9 and P10 retained ABA content similar to the noninfested plants (**Figure 5C**). Regarding the JA content, significant overaccumulation was detected in P1, P10 and P15, while in P2 and P14 it was depleted at 48hpi (**Figure 5B**).

Interestingly, in most accessions the JA and IAA (**Figures 5B, D**) content was below the limits of quantification (LOQs) of the method (**Supplementary Table 5**). In order to simplify the detailed distribution of accessions based on the trends of these fluctuations, the accessions were arranged in four distinct groups. More specifically, accessions with elevated JA abundance and reduced SA and ABA abundances form Group A. Accessions with increased JA, SA and ABA abundances are arranged in Group B. Group C is made of accessions with phytohormonal abundance similar to or less than that of noninfested plants. Group D is formed by accessions with decreased JA and ABA accumulation and increased SA accumulation. Details are provided online (**Supplementary Table 6**).

### 3.6 Untargeted profiling of secondary metabolites

**Figure 6:**
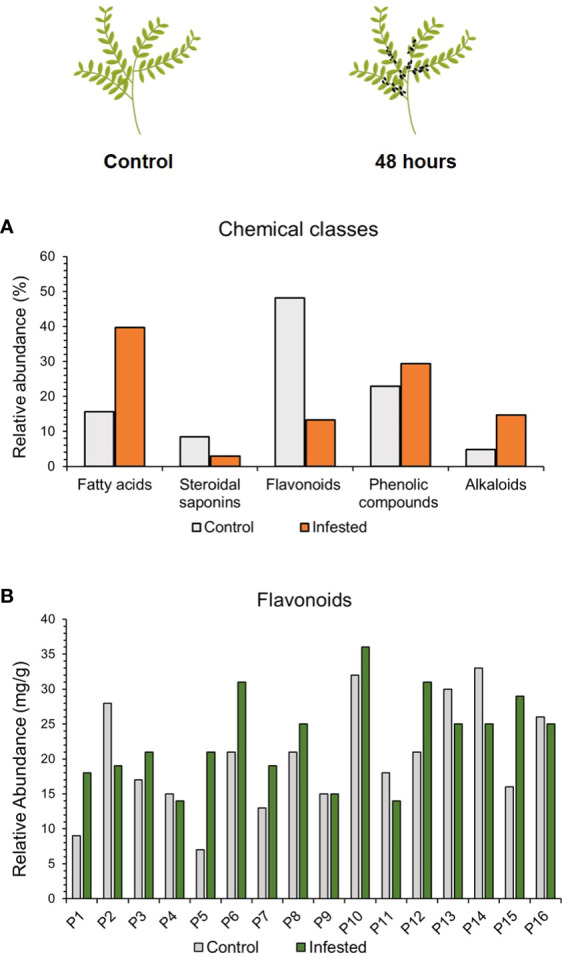
**(A)** Chemical classes contribution (%) to the metabolomic profile of lentil accessions and **(B)** semi-quantification of flavonoids (mg/g of dry weight) after 48h of aphid herbivory.

It is known that plants as sessile organisms use a large variety of secondary metabolites to defend against herbivores and pathogens. To investigate whether the effect of aphid infestation triggered a production of defense secondary metabolites the metabolomic profile of each lentil accession was analyzed after 48 hours post aphid herbivory in comparison to the untreated. The UHPLC-HRMS analysis, revealed changes in the presence of different compounds among the chemical classes of secondary metabolites, including flavonoids, fatty acids, alkaloids and saponins after 48hpi. Specifically, 40 flavonoids, 19 phenolic compounds, 13 fatty acids, 7 steroidal saponins and 4 alkaloids were detected prior infestation, whereas increased accumulation of 9 flavonoids, 20 phenolic compounds, 27 fatty acids, 2 steroidal saponins and 10 alkaloids were detected after aphid infestation (**Figure 6**).

Based on the PCA, a clear tendency of separation could be observed between the control and the infested samples (**Supplementary Figure 2**). These findings indicate that although some classes of metabolites, such as phenolic compounds seemed similar in abundance between the control and the infested samples there are significant differences at the composition and the abundance level of the compounds that need to be further investigated.

It should be noted that after aphid herbivory the total number of different flavonoid compounds decreased. Taking into account that HRMS metabolomics did not presuppose a quantitative approach, to demonstrate the differences in relative abundances the well-acknowledged flavonoids kaempferol and quercetin (aglycone forms) identified in all accessions, were used as surrogate compounds to extrapolate quantities and provide semi-quantitative results. More specifically, the quantitative flavonoid content was increased in 9 out of 16 accessions after aphid infestation (P1, P3, P5, P6, P7, P8, P10, P12, P15), whereas it remained similar to control in genotypes P4, P9 and P16.Intriguingly, a lower abundance of flavonoids was detected after infestation in genotypes P2, P11, P13 and P14 (**Figure 6B**). Analysis for specific compounds implicated in aphid-stress responses such as kaempferol (up to 2.4-fold accumulation), apigenin (2.2-fold accumulation) and rutin (1.9-fold accumulation) exhibited an increase after aphid herbivory, denoting a putative significant change in the flavonoid profile composition (data not shown) that should be furtherinvestigated.

Similarly, steroidal and triterpene saponins are specific to plant species and they are reported to be defense metabolites against aphids as well as after other insect herbivores (Sanchez-Arcos et al., 2019). In our study saponins content declined in aphid herbivory treated plants post 48h compared to control plants. However, this decrease appeared to be correlated to changes in saponin composition profile among control and aphid treated plants. Specifically, 7 distinct saponins were detected in the control samples while only 2 were discerned in treated plants at 48hpi. Triterpene saponins with similar chemical formulas to the triterpenoids Soyasaponin I and Pisumsaponin I, were discerned in treated plants, underlining a putative response to biotic stress that should be explored.

To further verify the observed differences, PLS-DA was carried out and showed a clear separation between control and infested samples after 48h of treatment (**Supplementary Figure 2**, ii). Additionally, the permutation testing secured the validity of the clustering in the two models, the treated (infested) and the control. Thus, variables for the treated/control with a ratio range of 0.067 to 1.5 and adjusted p-values ≤0.05 (Benjamini-Hochberg) were considered to contributing the most to group separation and were forward for annotation using *in house* and online databases (**Supplementary Figure 2**, iii).

Noteworthy that changes in secondary metabolites are not only detected at the abundance level but also at the profile composition level. Specifically untargeted metabolomics analyses revealed the participation of 86 distinct metabolites in the untreated control plants, while 68 were detected in aphid infested plants post 48h. Interestingly in certain groups of metabolites including alkaloids and fatty acids the number of compounds detected after aphid infestation was increased (**Supplementary Table 7**). While for the flavonoids and the saponins there was a decrease in the detected compounds. As for the phenolics group there was a slight increase in the metabolite compounds detected.

## 4 Discussion

Lentils represent a cool-season pulse crop of temperate zone grown mainly as a source of plant proteins, mineral and vitamins for human consumption. Lentil production is restrained by aphid infestation able to transmit a number of legume viruses with detrimental impacts on seed quality and crop yield. It is expected that farming systems may face new and intense pest problems in the coming years due to the changing climate. There is a serious risk of crop economic losses, as well as a challenge to global food security. To effectively secure crop production and food safety adaptive management strategies are required to deal with the crops’ resilience and the changing dynamics of pests. Thus, thorough understanding of plant-aphid interactions and molecular mechanisms regulating the lentil-aphid defense responses could provide valuable tools for breeding strategies as well as for pest management approaches.

In the present study, we evaluated the performance of lentil germplasm collection originating from Greece, to aphid infestation, and explored the contribution of four phytohormones to tolerance development, with special focus on the SA and JA transduction pathways and hallmark defense genes expression along with the profiling of secondary metabolism components after aphid herbivory.

The reduction of the aboveground fresh weight, dry biomass and shoot length are indicative of the plant’s nutritional imbalance due to aphid herbivory. These indices have already been used to distinguish between tolerant and susceptible lentil and vegetable germplasm (Ansari et al., 2018; Kumawat and Gothwal, 2018; Das et al., 2019; Mitku et al., 2019; Raeyat et al., 2021). In the present study, aphid infestation had diverse severity among the accessions, demonstrating no significant effect on accessions P8, and P10 dry weight, and on P8’s height. Interestingly, the commercial cultivar P16 performed better than most landraces, which exhibited limited ability of compensation to aphid induced damages. Accession P16 is a commercial cultivar and possibly breeding strategies have selected for traits that maintained plant performance and robustness to environmental adversities. In addition, the FW, DW and H as developmental indices are quantitative traits controlled by a number genetic loci and processes. Thus, no definite correlation was detected among the reduction of FW, DW, H and their combinations to the other defense parameters assessed. Similarly, the RWC indicator revealed the potential implication of defense mechanisms against sap efflux due to aphid infestation. Phloem-sap feeding aphids deplete nutrients from the plant altering its growth rate. Phloem-sap is generally free of toxins and feeding deterrents, thus the plant has to compensate for its depletion engaging the production of secondary metabolites to safeguard the plant cells’ osmotic potential and abort the intruders.

Antixenosis is an essential type of aphid resistance, as it can deter insect pests and delay the potential colonization in crops, before the induction of severe economic damages (Paudel et al., 2018). In comparison to previously featured antixenotic germplasm (Hesler, 2011; Tesfaye et al., 2013; Kordan et al., 2018; Mitku et al., 2019), we observed that most of the accessions hosted a small number of insects (less than 8), with five of them (namely P2, P3, P5, P10 and P13) being the least preferred, 48 hours after the release of *A. pisum*, indicating a considerable “non-preference” resistance mechanism, apparent in this germplasm. Chemical and vaporescent compounds of the plant tissues are responsible for the attraction or repulsion of insect pests. Contrary to biting-chewing insects’ mouth parts, the aphid’s stylet lacks gustatory chemoreceptors, resulting in incapability of volatile-organic-compound detection prior probing (Powell et al, 2006; Stec et al, 2021; Stec et al, 2021). Aphids can detect non-volatile chemicals on the plants cuticle after probing, using specific chemoreceptors located on the tips of their antennae (Ziv et al., 2018). Thus, the presence of less than 3 aphid individuals in the abovementioned accessions indicated a change in the cellular metabolites that deter aphids. However, induction and accumulation of secondary metabolites takes time to develop, underlining the reprogramming ability of plant defense mechanisms of detection and to combat invaders such as herbivorous insects.

The front line of plant defense responses to biotic and abiotic stress is the production of reactive oxygen species (ROS). ROS are actively produced as poisons to retort biotic intruders. Specifically hydrogen peroxide (H_2_O_2_) is primarily detected extracellularly, in the apoplast and cell walls of aphid-infested plants at early time points, followed by intracellular persistent cytosolic localization at later time points (Goggin and Fischer, 2022). Studies have shown that the pea aphid increased H_2_O_2_ and O_2_^−^ at 6 to 24 hours after aphid saliva infiltration in legume plants such as pea, faba bean and *Vicia sativa*. The H_2_O_2_ burst starting at 6hpi is maintained higher than in control plants until 48hpi, indicating its implication in signaling networks (Lukasik et al., 2017). Plant defenses against aphids in pea included also the implication of reactive nitrogen species nitric oxide (NO) with a role in plant–aphid interactions in conjunction with induction of hormone signaling pathways of SA, JA and ET (Arnaiz et al., 2021).

Hence redox signaling is pivotal for defense regulation, and is inherently linked to the cross-interference of phytohormones, including but not limited to SA, ABA, JA and IAA (Foyer et al., 2016). Phytohormones are an integral part of signal transduction pathways in plants granting plasticity to adapt to the ever changing developmental and environmental cues and orchestrate various processes that have significant contribution to development, growth, stress responses and their prioritization (Fichman & Mittler, 2020). Phytohormonal signaling is multiple and phytohormones may act both synergistically and antagonistically with one another (Aerts et al., 2021). Studies have shown that aphids in general induce the SA signaling pathway to deceive the plant defense because cross-talk between hormonal pathways impedes the SA-induced plant to fully induce the JA and ET pathways (Åhman et al., 2019). Interestingly aphids are more sensitive to the defense JA signaling pathway. Indications of this SA–JA antagonism have been found in the global expression studies of *A. pisum* on susceptible barrel medic (Yan et al., 2018). Based on this model the aphids induce the SA content and signaling pathway in all accessions except one (P1). Following, the JA content and signaling pathway were down regulated for most accessions except three, namely P1, P10 and P15 indicating a putative different pattern of SA/JA hormone cross-talk. Specifically, P1 had a low SA and high JA content compared to the control at 48h post aphid infestation. Also, this genotype appeared rather susceptible to aphids due to their preference, indicating possible differences at the genetic level as expression of hallmark genes of the JA signaling pathway is not distinct. Conversely, accession P10 with a 3fold increase of JA content after aphid infestation is sufficient to counteract the antagonistic interplay of SA/JA in favor of JA mediated defense responses, providing tolerance and resilience to aphids. While a lower JA content of 1fold increase compared to control in accession P15, but with a quite large increase of SA content was not able to alter the SA/JA antagonism setting the genotype susceptible to aphids.

Remarkably in this accession the putative role of ABA should not remain unnoticed. The ABA content in this accession is increased at 48h post aphid infestation compared to control. This increase is statistically significant and similar to that observed in accessions P5 and P11. However, P5 and P11 with similar SA content post aphid infestation did not possess any JA content, contrary to the P15 accession. These evidence provide corroborative reasons to consider that the explanation for aphid induced plant susceptibility via the SA/JA hormonal competition is somewhat simplified as previously indicated (Åhman et al., 2019). The ABA signaling in P5 and P11 results to aphid tolerance with P5 being the least preferred followed by P11 with lower ABA content. Recent studies in *Arabidopsis* demonstrated that the antagonistic effect of the SA and ABA on the JA pathway is mediated through cross-talk with the JA dependent genes, thereby modulating the JA‐induced defense responses to herbivory insects and resistance (Proietti et al., 2018).

Regarding the IAA levels, they were below detection levels in most infested and non-infested accessions. Interestingly, genotypes P5 and P10 presented considerable accumulation of IAA both prior and after aphid infestation, which was reduced in P5 after herbivory. Similarly to our results, the suppression of the auxin pathway during herbivory was previously reported, indicating provisional inhibition of plant growth and development as indicated here (Pandey et al., 2017). Comparable data were also demonstrated in the tomato-aphid interactions with *Macropsyphum euphorbiae* (Mbaluto et al., 2021). While Machado and co-workers (Machado et al., 2016) have shown that this auxin (IAA) is rapidly induced within the first 5 min of aphid attack in *Nicotiana attenuata* plants. IAA auxin is considered a rapid and specific signal that regulates a subset of systemic, JA-dependent secondary metabolites in herbivore-attacked plants. Indeed, at 48h post aphid herbivory the JA content was statistically higher only in the P1, P10 and P15 accessions, while remained unaltered to the rest accessions. The latter is in agreement with previous observations on tomato leaves after *M. euphorbiae* infestation while its relevance to the increased steroidal glycoalkaloid levels remained unclear (Mbaluto et al., 2021). Inversely in this study, the lentil germplasm exhibited a quite large increase of alkaloids content in response to aphid infestation, that could be partly justified by the JA-signaling. Studies of simulated herbivory in chickpea plants indicated that JA biosynthesis and accumulation is upregulated within the first 20 minutes of stress induction and coupled to upregulation of JA-signaling defense genes, as well (Pandey et al., 2017). Thus, the observed high JA content in the above accessions could be due to an over-accumulation of JA hormone or to continuous JA synthesis in those genotypes. However, a detailed temporal assessment of IAA and JA content in lentil-aphid interactions would further our understanding of hormone cross-talk as well as of alkaloids induction.

To indirectly monitor the activation of SA and JA signaling the *PR*-genes expression and proteins are frequently used in various plant-pathogen interactions. PR-1 proteins appear to possess anti-microbial activity and the PR-5 family are homologous to thaumatin- and osmotin-like proteins and exhibit destructive effects on the permeability of fungal plasma membranes. To date the only PR proteins studied in lentil are those of the PR-4 family that were shown to be upregulated in ascochyta blight infections. The antifungal activity of PR-4 proteins has been also shown in other plant-pathogen systems as well as in lentil (Sari et al., 2017).

Analysis of quantitative expression of *PR-1*, *PR-5*, *PR-4*, and *AOC* indicated that genotypes differed with respect to their activation of the SA and JA signaling pathways. Specifically, the SA signaling appeared to transiently induce expression of *PR-5* at 24hpi in all accessions and followed a decline at 48hpi, except for P9. *PR-1* induction followed a different pattern among accessions. At 24hpi *PR-1* expression was upregulated while at 48hpi it was further enhanced for accessions P3, P4 and P11, or declined in P15.

As for JA signaling hallmark genes, *PR-4* induction was upregulated at 24h followed by further increase at 48h. Expression of *PR* genes is downstream of SA and JA signaling cascades, with a time interval between activation and expression. This might explain the further 48h increase of *PR-4* induction compared to that of *AOC*. On the other hand, *AOC* induction was upregulated at 24h and transiently declined. Usually, *AOC* precedes the expression of *PR*-genes as a key enzyme of JA biosynthesis mediating plant defense responses to a wide range of biotic and abiotic stresses (Vick and Zimmerman, 1984). *AOC* genes compose a small gene family, with different numbers of *AOC* genes across plant species. Thus there are six *AOC* genes in soybean and three in *Lotus japonicus* (Li et al., 2019). Recent studies have shown that *AOC* genes are involved in regulation of multiple plant developmental processes including legume-mycorrhiza interactions (Isayenkov et al., 2005). In simulated herbivory studies in chickpea leaves *AOC* genes were upregulated following upregulation of JA accumulation within 20 minutes of stress induction (Pandey et al., 2017). In our study JA levels solely could not justify *AOC* induction for all accessions, as most of them do not exhibit JA content above control levels. Recent studies in watermelon have shown that *AOC* genes’ expression is upregulated by exogenous application of SA hormone within 9h and then decreased below control levels (Li et al., 2019). Thus, it is putative that the observed induction of *AOC* might be due to an interplay of JA and SA signaling. However, studies on SA/JA and ABA signaling in lentil and in legumes are limited to ascochyta blight and fungal infections and there is also a concept that pathogens and aphids are able to adjust plant hormone signaling to their advantage. Particularly it is suggested that *A. lentis* suppresses SA-mediated plant defense by deploying effectors. Similarly, effectors interfering with the SA pathway have also been identified in various types of plant pathogens (Sari et al., 2017). It is also known that legumes differ in their production of defense hormones depending on whether native or non-native pea aphid host races are feeding on plants (Sanchez-Arcos et al., 2019). Noteworthy that hormone differences might lead to changes in plant metabolomes, especially for compounds having a deterrent or toxic impact on aphids. However, the lentil-aphid interactions in this study contacted on the same legume species, lentil (*Lens culinaris* Medik.*)* under the same developmental stage and employing the identical pea aphid population suggests that determined differences are possibly due to genetic variations of molecular mechanisms that govern the lentil-aphid interactions and of knowledge gaps regarding the hormones cross-talk.

A substantial body of evidence, indicated that plants accumulate ROS in response to aphids, a damaging group of phloem-feeding insects. Plants respond to biotic stress by eliciting a SA-mediated hypersensitive reaction to limit pathogen spread and in the process *AOX* expression is enhanced. SA has been reported to inhibit mitochondrial electron transport chain (ETC) in tobacco cell cultures and this inhibition is thought to induce *AOX* expression. The AOX pathway regulates the cellular redox balance under biotic and abiotic stress, by diverting the cellular respiration and maintaining the redox homoeostasis of the plant cell (Polidoros et al., 2009). Expression of *AOX* is elicited by SA-signaling in low concentration while in high is aborted (Saha et al., 2016). Also, SA signaling inhibits the electron transport chain in mitochondria. Results showed that in the lentil-aphid interactions the *AOX* expression remained suppressed in all accessions and there is no apparent induction in response to the SA levels observed in accessions at 48h post aphid infestation. Studies in legumes (Mhadhbi et al., 2013; Zhang et al., 2012) have shown that AOX provides resistance to biotic stress induced by pierce-sucking insects, while relatively little variation in expression levels have been detected in legumes in response to biotic stress (Sanchez-Arcos et al, 2019; Sweetman et al., 2019). Thus, it is possible that aphids as phloem feeding insects do not induce a hypersensitive response of the host-plant, ensuring their survival.

Due to their immobile life status plants have evolved cross-linked networks to sense, perceive and respond to environmental adversities. ROS and hormone signaling are also implicated in the production of secondary metabolites under biotic and abiotic stress responses. Studies have shown that IAA promotes the production of phenolics and flavonoids, in a dose-dependent induction as was observed in *N. attenuata* tissue cultures (Machado et al., 2016). In this study total phenolics percentage increased at 48h post aphid herbivory, although IAA levels were decreased. IAA auxin is considered a rapid and specific signal that regulates a subset of systemic, JA-dependent secondary metabolites in herbivore-attacked plants. JA-responsive transcription factors were implicated in the production of phenolic acids and alkaloids in various plant species (Zhan et al, 2022 and references therein). Aphid-infestation induced the biosynthesis and accumulation of alkaloids in lentil accessions. Remarkably aphid infestation induced also the production of new alkaloid compounds in lentil in comparison to control. A number of alkaloids are known to possess toxicity against various chewing insects as well as sucking insects such as aphids and whiteflies (Bhambhani et al., 2021). Induction of alkaloids was also observed in lupin species in response to aphids.

Phloem-sap in general is a poorly-defended nutrient rich source of food for herbivorous insects (Douglas, 2006). Thus its armoring with toxic metabolic compounds renders invaders restriction. Alkaloids are also known for their antimicrobial activity against various pathogens including fungi, bacteria and some viruses (Bhambhani et al., 2021) and to various biotic challenges, such as in attacking insects, herbivores, and pathogens; thus, protecting the plants against a range of biotic adversities.

Interestingly the most frequent class of unique chemical compounds identified in the pea aphid host plants studied was the flavonoids (Sanchez-Arcos, et al., 2016; Sari et al, 2017; Stec et al, 2021). Flavonoids, particularly isoflavonoids, are especially abundant in legumes. Indeed the flavonoid content in lentil prior to aphid-infestation is the highest in comparison to other secondary metabolites. It is known that flavonoids are a major class of phenolics with antioxidant activity implicated in the removal of ROS. However, upon aphid infestation the total number of different lentil flavonoids was decreased, in spite of an observed increase in certain flavonoid compounds such as kaempferol, apigenin and rutin. Kaempferol is an antioxidant flavonoid involved in the redox homeostasis of the plant cell. Recent studies have shown that kaempferol and apigenin treated pea plants exhibited a decrease in the intensity of aphid-sap ingestion, although treatment with these flavonoids did not prohibit aphid attack. Also kaempferol made the finding of sap-transporting vessels more difficult for aphids, underlining a protection role to biotic stress (Stec et al, 2021). Interestingly kaempferol together with quercetin glycosides such as rutin are found in the aerial parts of lentil plants, where the later compounds represent about 73% of the total determined flavonoids. In fact, rutin is considered the most abundant flavonoid in the plant kingdom (Dias et al., 2021) and quercetin glycosides are thought to play a central role in the cellular redox balance under stress adversities. Flavonoids are regarded as secondary antioxidants that complement the primary antioxidants upon large alterations in redox homeostasis that depress the activity of the later while promoting the biosynthesis of the flavonoid antioxidants (Agati et al., 2020). Noteworthy that such flavonoids were previously shown to be linked to resistant genotypes (Togola et al., 2020; Stec et al, 2021) indicating an innate biotic stress response to aphid infestation. However what molecular mechanisms and chemical signals elicit this response remains to be explored.

Saponins are distributed in many plant families, including legumes and have been reported to play a prevalent role in resistance against insect herbivores. The genus *Medicago* is well known for containing a complex mixture of triterpene saponins with a broad spectrum of biological properties including resistance against aphids as in *Medicago sativa* (Sanchez-Arcos et al., 2019). Saponins exhibit strong detergent properties, and can interact with membranes disturbing permeability and leading to cell death and necrosis. The saponins content was downregulated in response to aphid infestation in lentil accessions. This decrease in saponins could possibly explain partly the increased performance and preference of pea aphid to lentil accessions.

Fatty acids (FAs) are major and essential constituents of plant cells, providing structural integrity and energy for various metabolic processes. Fatty acid biosynthesis is subject to hormonal regulation, and their yield is critical in improving the plants’ ability to deal with multiple stresses (He et al., 2020; Cavaco et al., 2021). Interestingly, FAs and lipids can function as signal transduction mediators both intracellular and extracellular. FAs in the unsaturated form can serve as intrinsic antioxidants reacting and consuming ROS, while their oxidation gives rise to products implicated in the JA-signaling and ROS modulation and signaling (Cavaco et al., 2021). Fatty acids content increased in response to aphid infestation in lentil germplasm and also new compounds were induced indicating a pivotal role in lentil-aphid interactions. FAs are used as a reserve of carbon and energy source, taken into account that the phloem-sap feeders deplete the plant of photosynthates, restricting its growth, an increase in FAs content could replenish this depletion.

**Figure 7:**
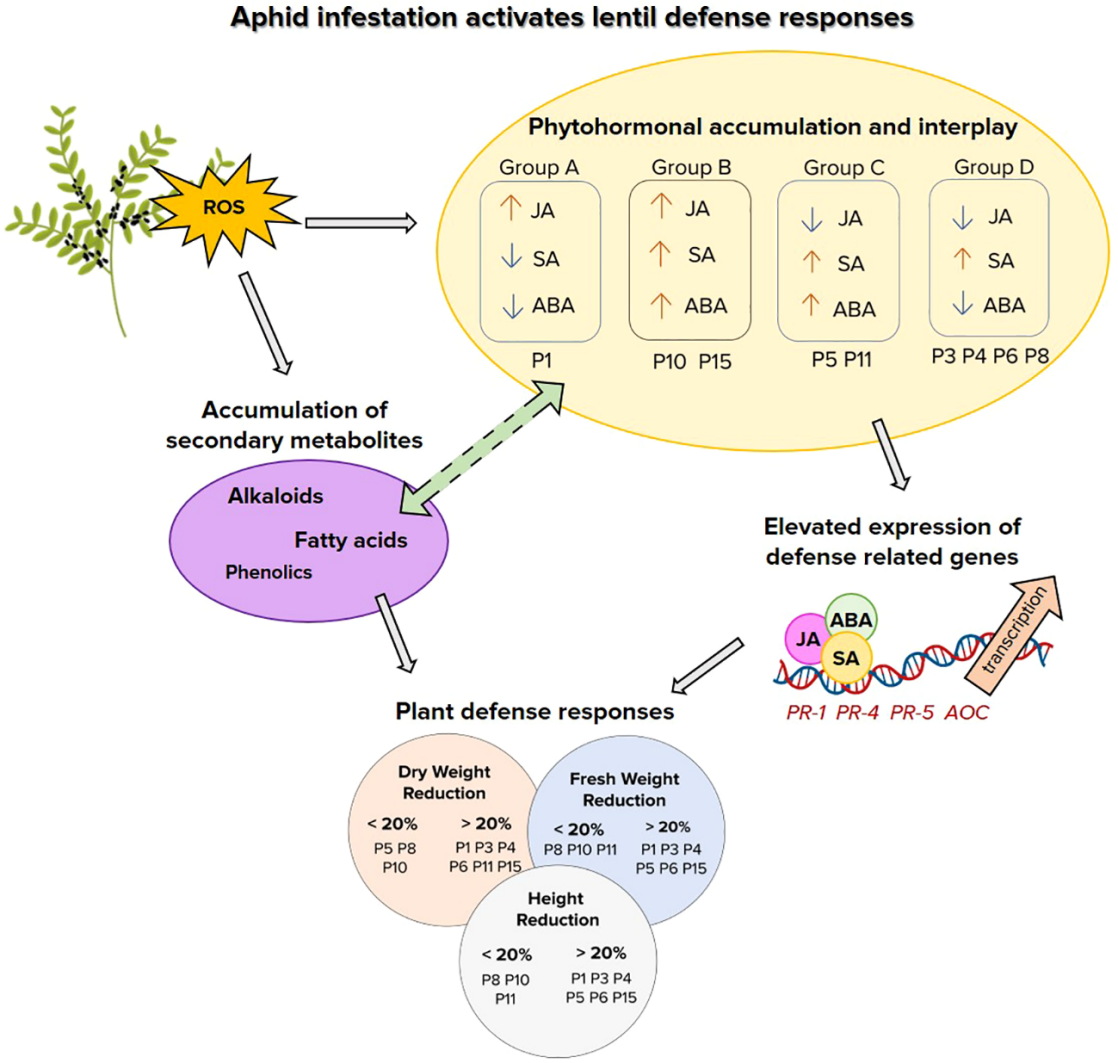
Schematic representation of lentil-aphid interactions and defense signaling pathways. Reactive oxygen species (ROS), jasmonic acid (JA), salicylic acid (SA), abscisic acid (ABA). Pathogenesis related genes *PR-1, PR-4, PR-5* and allene oxidase cyclase *(AOC).*.

Recent studies have shown that FAs and unsaturated forms are utilized as raw material to produce numerous aliphatic compounds, including membrane glycerolipids, cutin/suberin, nitroalkenes and jasmonates. The latter highlights the pivotal role of JA signaling in plant-pathogen interactions mediated through FAs crosstalk. In stress response JA signaling interplays with other signaling molecules either synergistically or antagonistically (Wang et al., 2020). Specifically, upon mechanical wounding JA biosynthesis increases production of H_2_O_2_ and antioxidant enzymes. Inversely H_2_O_2_ can upregulate JA biosynthesis genes. While the interplay of SA and ABA in this cross-talk is still ambiguous, depending on the pathogen and the host-plant interactions explored (Cavaco et al., 2021). It is reported, that aphids are more prone to JA-redox-related defense mechanisms (Selig et al., 2016), and induce the expression of genes related to the antagonistic SA pathway (Åhman et al., 2019), that is also confirmed in this study. While the increased accumulation of FAs in lentil-aphid interactions unraveils a significant research area of hormone signaling and metabolome reprogramming of plant defense. A schematic respresentation of lentil defense responses to aphid infestation is depicted in **Figure 7**.

In conclusion, lentil-aphid interactions are governed by an interplay of ROS and hormone signaling for induction of defense responses and secondary metabolites. While defense genes induction underscored the role of the SA signaling that is effective against biotrophs, though the importance of JA signaling and ABA/SA cross talk highlighted the induction of early defense responsive genes and possibly of secondary metabolites. These could be used to monitor the plant defense responses during the first hours of aphid attack and capture the role of hormones cross talk and ROS signaling in in association to metabolome reprogramming. The induction of alkaloids, phenolics and fatty acids and that of specific flavonoids provide valuable tools for further germplasm screening and breeding strategies, as the development of insect-resistant cultivars having a heritable and transferable resistance is the most sustainable alternative to foster food security in a climate changing environment. Apparently lentil resilience to aphid herbivory is attributed to a cascade of phytohormonal crosstalk induced probably by ROS during stress, which further activates defense responses through involvement of defense genes and biosynthesis of defense-related secondary metabolites. Further research could shed light on these particular interplay to unravel key components that orchestrate and govern lentil insect resilience.

## Data availability statement

The original contributions presented in the study are included in the article/**Supplementary Material**. Further inquiries can be directed to the corresponding author.

## Author contributions

Conceptualization: PM. Methodology: IZ, SN, EB, KK, TB, KM and PM. Project administration: PM. Investigation: IZ, SN, EB, KK, TB and KM. Funding acquisition: PM. Formal analysis: IZ, SN, EB and KK. Resources: KM and PM. Writing – original draft: IZ and SN. Writing – review & editing: IZ and PM. All authors have read and agreed to the published version of the manuscript. All authors contributed to the article and approved the submitted version.

## Supplementary material

Supplementary Figure 1 Two-dimensional PCA of 16 lentil accessions according to the relative gene expression of *AOX*, *PR-1*, *PR-5*, *PR-4* and *AOC* genes. The first two axes represent 60.16% of the observed variation.

Supplementary Figure 2 PCA (Ai, Bi), PLS-DA (Aii, Bii) score plots and permutation tests (Aiii, Biii) from the comparison of lentil samples after 48 h of treatment (red) with control samples (black) employing UHPLC-HRMS analysis in negative **(A)** and positive **(B)** ion modes, respectively. The goodness of fit and prediction of the models were for PCA analysis (Ai) R2X=0.637, (Bi) R2X= 0.63 and for PLS-DA analysis (Aii) R2X = 0.286, R2Y=0.884, Q2 = 0.524, (Bii) R2X = 0.24, R2Y=0.932, Q2 = 0.627.
