# Supplementary figures and images for: Ingenious characterization and assessment of lentil germplasm collection to aphid Acyrthosiphon pisum stress unveils distinct responses
Source: Front Plant Sci. 2022 Dec 21;13:1011026. doi: 10.3389/fpls.2022.1011026 (PMC9811392; doi:10.3389/fpls.2022.1011026)

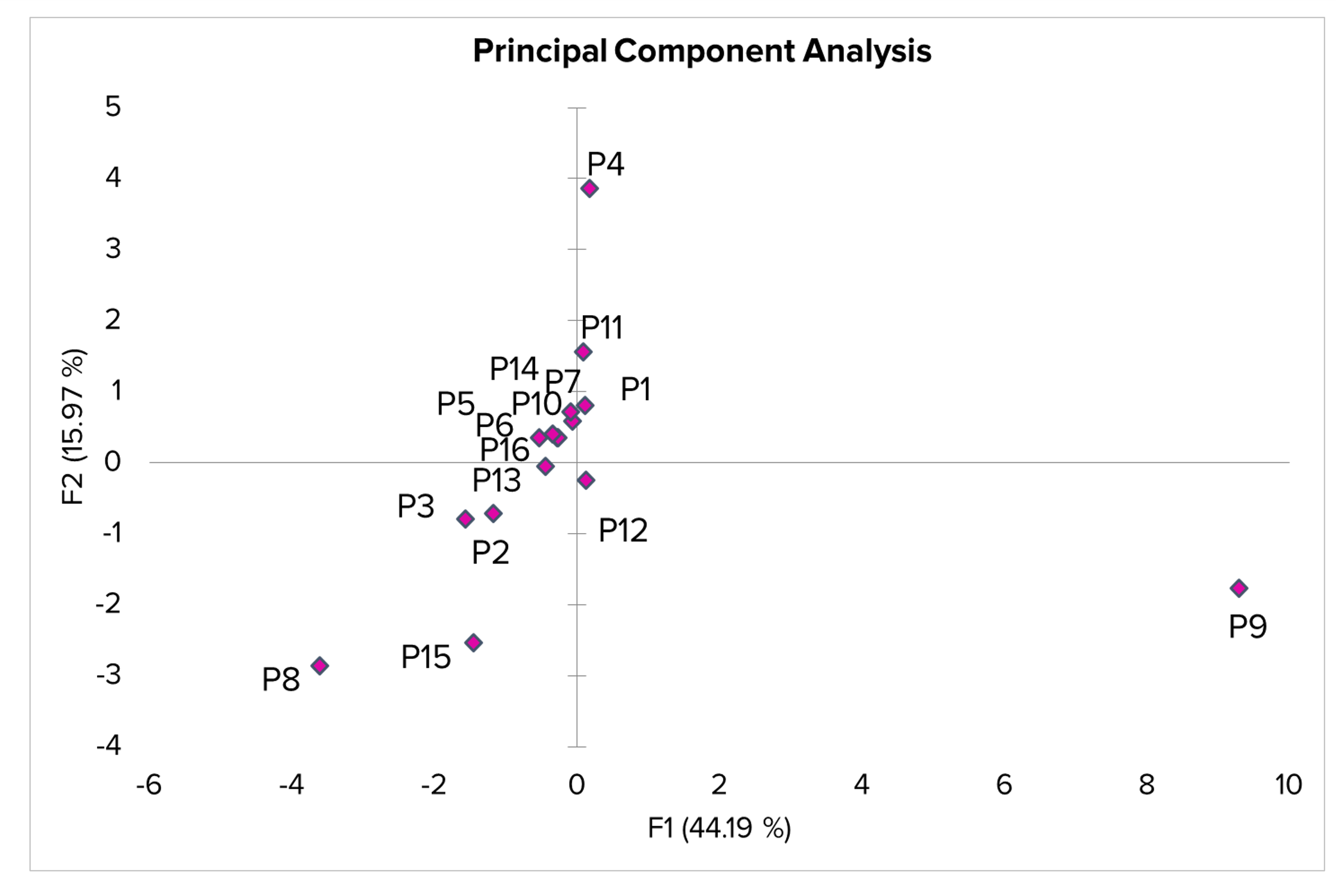

Supplement: Supplementary Figure 1 — Two-dimensional PCA of 16 lentil accessions according to the relative gene expression of AOX, PR-1, PR-5, PR-4 and AOC genes. The first two axes represent 60.16% of the observed variation. [file Image_1.jpeg]

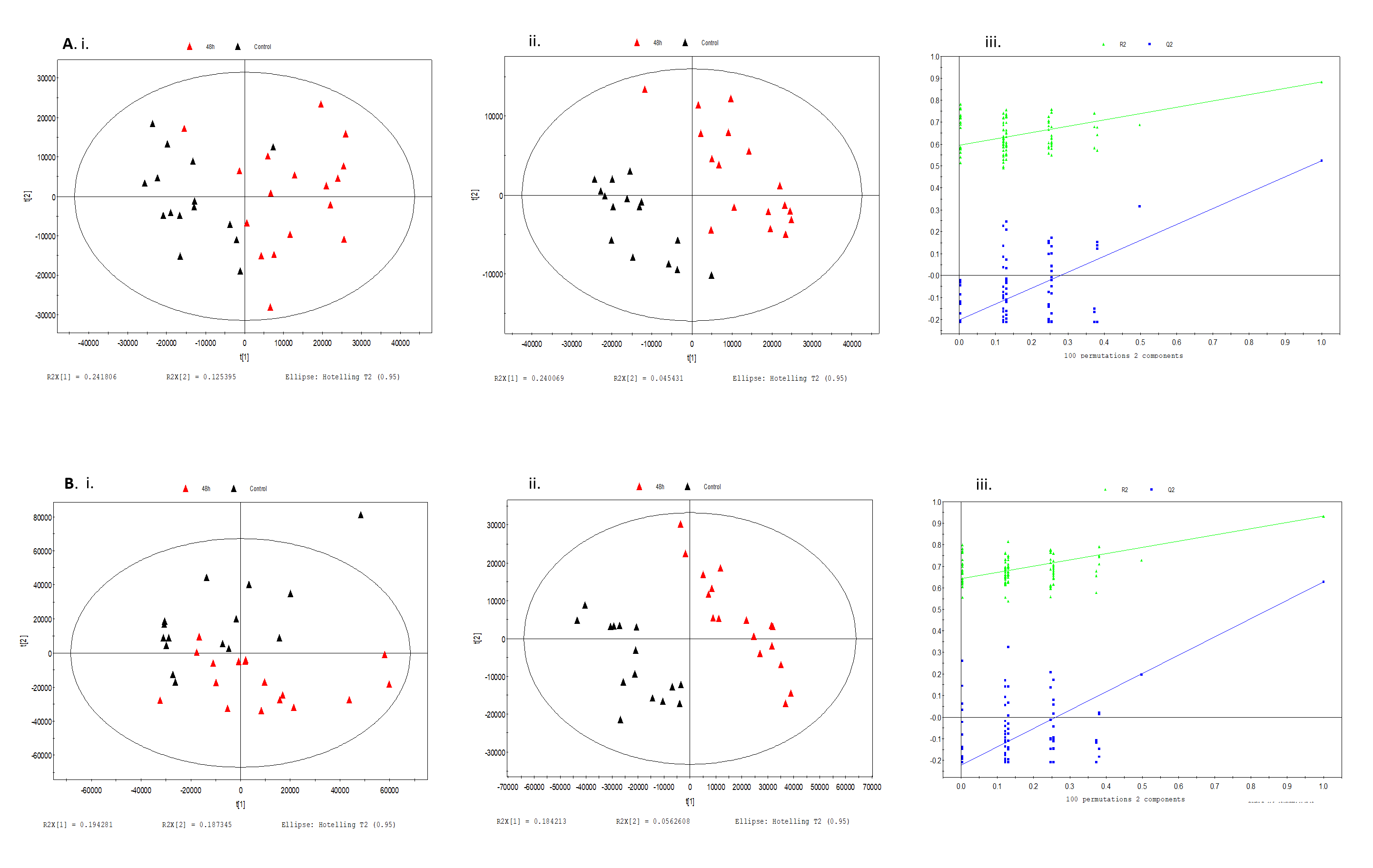

Supplement: Supplementary Figure 2 — PCA (Ai, Bi), PLS-DA (Aii, Bii) score plots and permutation tests (Aiii, Biii) from the comparison of lentil samples after 48 h of treatment (red) with control samples (black) employing UHPLC-HRMS analysis in negative (A) and positive (B) ion modes, respectively. The goodness of fit and prediction of the models were for PCA analysis (Ai) R2X=0.637, (Bi) R2X= 0.63 and for PLS-DA analysis (Aii) R2X = 0.286, R2Y=0.884, Q2 = 0.524, (Bii) R2X = 0.24, R2Y=0.932, Q2 = 0.627. [file Image_2.jpeg]
